# Inhalable Dry Powder of Bedaquiline for Pulmonary Tuberculosis: In Vitro Physicochemical Characterization, Antimicrobial Activity and Safety Studies

**DOI:** 10.3390/pharmaceutics11100502

**Published:** 2019-10-01

**Authors:** Mohammad A. M. Momin, Bhamini Rangnekar, Shubhra Sinha, Chen-Yi Cheung, Gregory M. Cook, Shyamal C. Das

**Affiliations:** 1 School of Pharmacy, University of Otago, Dunedin 9054, New Zealand; mammomin@vcu.edu (M.A.M.M.); bhaminirangnekar@gmail.com (B.R.); shubhra.sinha@otago.ac.nz (S.S.); 2 Department of Pharmaceutics, School of Pharmacy, Virginia Commonwealth University, Richmond, VA 23298-0533, USA; 3 Department of Microbiology and Immunology, University of Otago, Dunedin 9054, New Zealand; chen-yi.cheung@otago.ac.nz (C.-Y.C.); greg.cook@otago.ac.nz (G.M.C.)

**Keywords:** bedaquiline, inhalation, formulation, spray-drying, dry powder, tuberculosis

## Abstract

Bedaquiline is a newly developed anti-tuberculosis drug, conditionally approved by the United States Food and Drug Administration (USFDA) for treating drug-resistant tuberculosis in adults. Oral delivery of bedaquiline causes severe side effects such as increased hepatic aminotransferase levels and cardiac arrhythmias (prolongation of QT-interval). This study aimed to develop inhalable dry powder particles of bedaquiline with high aerosolization efficiency to reduce the side-effects of oral bedaquiline. Bedaquiline (with or without l-leucine) powders were prepared using a Buchi Mini Spray-dryer. The powders were characterized for physicochemical properties and for their in vitro aerosolization efficiency using a next-generation impactor (NGI). The formulation with maximum aerosolization efficiency was investigated for physicochemical and aerosolization stability after one-month storage at 20 ± 2 °C/30 ± 2% relative humidity (RH) and 25 ± 2 °C/75% RH in an open Petri dish. The cytotoxicity of the powders on A549 and Calu-3 cell-lines was evaluated using the 3-(4,5-dimethylthiazol-2-yl)-2,5-diphenyltetrazolium bromide (MTT) assay. The powders were also evaluated for antimicrobial activity against *Mycobacterium tuberculosis.* The aerodynamic diameter of the l-leucine-containing powder was 2.4 µm, and the powder was amorphous in nature. The aerosolization efficiency (fine-particle fraction) of l-leucine-containing powder (fine-particle fraction (FPF): 74.4%) was higher than the bedaquiline-only powder (FPF: 31.3%). l-leucine containing powder particles were plate-shaped with rough surfaces, but the bedaquiline-only powder was spherical and smooth. The optimized powder was stable at both storage conditions during one-month storage and non-toxic (up to 50 µg/mL) to the respiratory cell-lines. Bedaquiline powders were effective against *Mycobacterium tuberculosis* and had a minimal inhibitory concentration (MIC) value of 0.1 µg/mL. Improved aerosolization may help to combat pulmonary tuberculosis by potentially reducing the side-effects of oral bedaquiline. Further research is required to understand the safety of the optimized inhalable powder in animal models.

## 1. Introduction

After acquired immunodeficiency syndrome (AIDS), tuberculosis (TB) is the leading cause of infectious disease deaths worldwide. It is estimated that in 2017, more than 1.3 million people died of TB, 10.0 million people developed TB disease, and about 23% (1.7 billion) of the world’s population is at risk of developing active TB [1]. The epidemic situation of global TB has been aggravated by the rapid emergence of drug-resistant TB where the causative microorganism of TB, *Mycobacterium tuberculosis,* shows resistance to multiple first-line drugs (e.g., isoniazid and/or rifampicin) or sometimes those cases are untreatable by the available anti-TB drugs [2]. Development of new drugs could be an alternative option to improve the current situation of global TB [3].

The United States Food and Drug Administration (USFDA) approved a new anti-TB drug, Bedaquiline or TMC207, in 2012 for the treatment of drug-resistant TB in adults when no alternative treatment option is available [4,5,6]. The preclinical evaluation of bedaquiline alone and in combination with other anti-TB drugs (e.g., moxifloxacin and pyrazinamide) using a murine model of TB indicated the potential of the use of bedaquiline alone and in combination for the treatment of TB [4,7,8]. Clinical studies were conducted on 47 multidrug-resistant TB patients, using bedaquiline or placebo, both in combination with five-drug, second-line anti-tubercular treatment regimen [9,10,11,12]. In these studies, the doses of bedaquline were 400 mg once daily for two weeks, then 200 mg three times weekly for 6 or 22 weeks. The addition of bedaquiline to the regimen resulted in faster culture conversion in those studies, suggesting that treatment time with the bedaquiline regimens will be shorter than other regimens However, in these studies, there were more deaths in the bedaquiline group than the placebo group. Also, along with the most common adverse effects (nausea, arthralgia and vomiting), more severe adverse effects such as increased hepatic aminotransferase levels and prolongation of QT-interval were also observed in the oral bedaquiline group [11,13]. Thus an alternative delivery which can potentially reduce the adverse effects of current oral delivery should be explored.

Pulmonary drug delivery has been used for many years for the treatment of lung diseases such as asthma and chronic obstructive pulmonary disease (COPD), and it has the potential to improve the treatment of TB [14,15]. The dry powder inhaler (DPI) is the most attractive pulmonary drug delivery system which has several advantages over oral and parenteral treatment [16,17]. The success of DPI for TB depends on the production of powders with favorable aerodynamic properties which are highly aerosolizable and capable of delivering the maximum amount of drug to the deep lungs [18].

Spray-drying is an attractive and well-established particle engineering technique with its manipulation capacity and scalability to produce inhalable dry-powder particles with desired aerodynamic properties [19,20]. However, spray-drying of drug-only formulation often results in low process yield and poor aerosolization. These problems can be resolved by adding amino acids in the feed solution [21,22,23,24]. Among the amino acids, l-leucine has been widely used in the formation of dry powder particles, improvement of process yield and aerosolization of spray-dried powders [25,26,27,28]. It is generally regarded as a safe excipient for pulmonary delivery.

Recently chitosan-based dry powder bedaquiline nanoparticles were produced using freeze drying where inhalable dry powders were produced by mixing with lactose pre-blend [29]. This report is mainly a carrier-based formulation limiting its ability to deliver a high dose. Also, nanoparticles are not a good choice for deep lung delivery since a large fraction of these particles (< 1 μm) is exhaled from the respiratory system [30]. Moreover, freeze-drying is not a feasible technique for powder production since it has limited control over particle-size distribution and it produces low-dispersibile powders [31]. Currently, there is no DPI formulation of bedaquiline microparticles although it has a promising therapeutic benefit. Bedaquiline is a new drug, and it has substantially different physicochemical properties to the drugs reported earlier by our group [28,32,33,34,35]. It was not known whether it was feasible to prepare inhalable bedaquiline microparticles which will have high aerosolization capacity. The current study aimed to produce inhalable bedaquiline microparticles with high aerosolization performance by spray drying to use for treating tuberculosis in order to reduce the adverse effects associated with the existing oral bedaquiline. l-leucine (20% *w/w*) was used in the formulation to improve the process yield and aerosolization. Previously it has been reported that l-leucine improves the aerosolization and physical stability of DPI formulations which are related to the surface enrichment of l-leucine on particles and intermolecular interactions with the drugs [36,37]. l-leucine also decreases surface-free energy which contributes to improved aerosolization [38]. Powders were investigated for physicochemical properties (particle size, morphology, crystallinity and drug content), in vitro aerosolization performance, and physical stability during storage. The powders were also investigated for cytotoxicity on respiratory cell lines to check the suitability for inhalation delivery and for antimicrobial activity against *Mycobacterium tuberculosis.*

## 2. Materials and Methods

### 2.1. Chemicals and Reagents

Bedaquiline (purity: 99.5%) and l-leucine (purity: 98.0%) were purchased from DC Chemicals, (Shanghai, China and Hangzhou Dayangchem Co., Ltd., Hangzhou, China). High-performance liquid chromatography (HPLC)-grade organic solvents (methanol and ethanol) were from Merck, Darmstadt, Germany; analytical reagent (AR) grade ammonium formate, formic acid and silicone oil (viscosity 10 cSt) from Sigma–Aldrich (St. Louis, MO, USA). Water was collected from Milli-Q water purification system (Millipore Corporation, MA, USA) and filtered through 0.45 μm membrane filter (Phenomenex, CA, USA) before use.

### 2.2. High-Performance Liquid Chromatography (HPLC) Analysis

Bedaquiline was quantified using a validated HPLC method. Briefly, the HPLC system was equipped with an LC-20AD pump, SPD-M20A prominence detector, DGU-20A5 degasser, SIL-20AC prominence auto sampler and Class-VP 7.4SP4 software (Shimadzu, Japan). Chromatographic separation was achieved on a Synergi Fusion RP 80A C_18_ column (4 µm, 150 × 4.6 mm) protected by a C18 guard column (4.0 × 3.0 mm) (Phenomenex, Torrance, CA, USA).

The mobile phase consisted of ammonium formate buffer, pH 6.3 and methanol (2:98% *v/v*) at a flow rate of 1.0 mL/min. The injection volume was 20 µL, and the run time was 6 min. Bedaquiline was detected at a wavelength of 249 nm. The calibration curve for bedaquiline was linear (*R*^2^ > 0.999) over the concentration range of 2–100 µg/mL. The accuracy (%bias) and precision (%coefficient of variation, CV) were within acceptable limits (≤ 15%) [39,40].

### 2.3. Preparation of Powders

Bedaquiline powders (with or without 20% *w/w* l-leucine) were produced using a Buchi B-290 Mini Spray-Dryer (Buchi Labortechnik AG, Flawil, Switzerland) with a high-performance cyclone in a closed-mode using nitrogen as the atomizing gas. Briefly, feed solutions (0.5% *w/v*) were prepared by dissolving the formulation components in a co-solvent system of ethanol and water (90:10, *v/v*) with the aid of sonication and moderate shaking. Feed solutions were spray-dried under the following operating conditions: feed rate 2 mL/min, spray gas flow rate 670 L/h, inlet temperature 70 °C, aspiration rate 50% and the two-fluid nozzle diameter 0.7 mm. The resulting outlet temperature was 47–53 °C at the above set of operating conditions. l-leucine was added in the current formulation to maximize the process yield and aerosolization efficiency while the concentration of l-leucine was kept low to keep the drug loading high in the powder. After spray drying, the powders were put into screw-capped glass scintillation vials, assessed for process yield and stored in a desiccator at room conditions until used for studies. The process yields of the collected powders were calculated as a percentage mass of the powder obtained compared to the initial mass of the solids dissolved in the feed solution.

### 2.4. Estimation of Drug Content

Approximately 5 mg of spray-dried powder (triplicate of each powder sample) was dissolved in 100 mL of methanol and analyzed for the drug content by the HPLC method mentioned above (Section 2.2).

### 2.5. Minimal Inhibitory Concentration (MIC) against M. Tuberculosis

The minimal inhibitory concentrations (MICs) of bedaquiline and bedaquiline with L-leucine against *Mycobacterium tuberculosis* mc^2^6230 were determined as previously described [41]. Strain mc^2^6230 is an avirulent auxotrophic *M. tuberculosis* mutant. When grown in pantothenate-supplemented medium, it behaves like wild-type *M. tuberculosis*. However, it is not capable of causing disease even in severe combined immunodeficiency (SCID) mice [42,43]. *M. tuberculosis* mc^2^6230 was grown in Middlebrook 7H9-oleic acid-albumin-dextrose-catalase (OADC)-tyloxapol and plated on solid 7H11-OADC at 5% CO_2_ and 20% O_2_, which were supplemented with pantothenate (50 µg/mL). Briefly, cell inoculum was diluted with 7H9 medium to achieve a final OD_600_ of 0.05, transferred to wells of a 96-well microtitre plate. Then the drug formulations were dissolved in dimethyl sulfoxide (DMSO), diluted to appropriate concentrations (two-fold dilutions over the range 0.02–10 µg/mL) and added in 7H9 medium. Following incubation at 37 °C for 5 days, 0.02% resazurin solution was added to the wells and incubated at 37 °C for 24 h. The MIC defined as the lowest concentration of the formulations able to completely inhibit bacterial growth. All experiments were performed in biological triplicate.

### 2.6. Morphology and Particle Size

The morphology and particle size of the spray-dried powders were determined following our previous report [44]. Briefly, samples were mounted on a sterilized metal stub and sputter coated with a gold/palladium alloy (10 nm thick) using a K575X sputter coater (EM Technologies Ltd., Kent, England). The visual images of the powder samples were captured using a scanning electron microscope (JEOL Ltd., Tokyo, Japan) at an accelerating voltage of 5 kV. The mean geometric diameter (*n* ≥ 250) of the spray-dried powder particles was determined from the scanning electron microscope (SEM) images collected at different magnifications using ImageJ 1.48 software (ImageJ, NIH, USA).

### 2.7. Thermal Analysis

#### 2.7.1. Differential Scanning Calorimetry

Thermal properties of supplied bedaquiline, l-leucine and spray-dried powders of bedaquiline (with or without l-leucine) were analysed using differential scanning calorimetry (DSC Q100, TA Instruments, New Castle, DE, USA). About 5 mg of powder sample was sealed in an aluminium pan and scanned over a range of 25 °C to 300 °C at 10 °C/min under nitrogen environment (at purge of 40 mL/min). An empty (without sample) sealed pan was used as a reference. Data were analysed using TA Universal Analysis 2000 software (TA Instruments, New Castle, DE, USA).

#### 2.7.2. Hot-Stage Microscopy

Hot-stage microscopic studies of the supplied and spray-dried powders were conducted using a phase contrast light microscope (Nikon Optiphot PFX, Tokyo, Japan) fitted with a polarizer. The system was equipped with a Mettler Toledo FP90 central processor and a hot stage (Mettler Toledo FP82HT, Zurich, Switzerland). Briefly, the powder sample was mounted on a glass slide with a cover slip and heated over a range of 25 °C to 300 °C at 10 °C/min. The heating program was controlled by Image Pro Plus software (version 7.0) and images were captured via OPTIKAM PRO5 digital camera (OPTIKA SRL, Ponteranica, Italy) at a magnification of 200X.

### 2.8. Crystallinity

The crystallinity of the supplied and spray-dried powders was conducted using an X-ray powder diffractometer (PANalytical X’Pert PRO MPD PW3040/60 XRD, Almelo, Netherlands). The system is equipped with a Cu-Kα radiation source generated at 40 kV and 40 mA and a rapid real time multi-strip X’Celerator detector. Powder sample was loaded in an aluminium sample holder as a thin layer and scanned over a 2θ range of 5–35° at 2°/min under ambient conditions. PANalytical High Score software (version 4.0) was used to process and analyze the data.

### 2.9. Drug-Excipient Interaction

The structural change or interactions between formulation components in the spray-dried samples were analyzed using a Varian 3100 Fourier transform infrared (FTIR) (Varian Inc., Palo Alto, CA, USA) equipped with an attenuated total reflectance (ATR) (Gladi ATR, Madison, WI, USA) accessory. The powder sample was placed on the diamond ATR crystal and scanned (total 32 scans) at 4 cm^−1^ spectral resolution over the range of 500 to 4000 cm^−1^. The data was collected and analysed using the Varian Resolutions software (version-5.2.0 CD 846).

### 2.10. In Vitro Aerosolization

The aerosolization behaviour of the spray-dried powders was evaluated by a next-generation impactor (NGI) with a mouthpiece adaptor and induction port (Copley Scientific Ltd., Nottingham, UK). Prior to aerosolization, all the NGI cups were coated with silicone oil to avoid particle bounce. Powder sample (20 mg) was loaded into a size 3 hard gelatin capsule (Capsugel, Tokyo, Japan) and placed into the aerolizer device (Novartis Pharmaceuticals UK Ltd., Surrey, UK). Powder was released from the device into the NGI at a flow rate of 100 L/min for 2.4 s. The rate of air flow through the NGI was adjusted using a digital flow meter (Copley Scientific Ltd., Nottingham, UK). The cut-off diameters of stages 2 to 7 at 100 L/min were 3.42, 2.18, 1.31, 0.72, 0.40 and 0.24 µm, respectively. Generally, the flow rate of 60 L/min or 28.3 L/min can be used for patients. However, we used 100 L/min since for a low-resistant device such as the aerolizer [45]; one should use 100 L/min flow rate for 2.4 s to get an inhalation volume of 4 L which is considered as the normal forced inhalation capacity of an average sized male. A similar flow rate for the aerosolization study of anti-TB powder formulations using the same device has already been reported [46,47]. Triplicate samples were prepared for the aerosolization performance of each powder formulation. Powders retained on the aerolizer device along with the capsule and the powder deposited in the mouthpiece adaptor (MP), induction port (IP) and all NGI stages (1 to 7 and micro-orifice collector, MOC) were collected using methanol and analyzed by the HPLC method mentioned in Section 2.2.

The emitted dose (ED, %) and fine-particle fraction, FPF (%) were calculated as reported previously [44]. Briefly, ED (%) is the percentage of ED (drug discharged from the capsule and device) relative to the total recovered dose (total mass collected from the device, capsule shell, MP, IP and all NGI stages); FPF (%) is the percentage of fine particle dose (total drug deposited from stages 2 to MOC) relative to the ED. The mass median aerodynamic diameter (MMAD) and geometric standard deviation (GSD) were calculated from the NGI data using the Copley inhaler testing data analysis software (CITDAS 3.10).

### 2.11. Cytotoxicity

Cytotoxicity of the bedaquiline powder (with or without l-leucine) on A549, alveolar basal epithelial cell-line and Calu-3, bronchial epithelial cell-line (ATCC, Rockville, MD, USA), was conducted following the procedure mentioned previously [28]. Briefly, A549 and Calu-3 cells were cultured using 1% (*v/v*) antibiotic-antimycotic solution and seeded onto sterile 96-well plates. The cells were treated with different concentrations (1 to 150 µg/mL) of bedaquiline in complete F12K (for A549 cells) and eagle’s minimal essential medium (EMEM) (for Calu-3 cells) with a final volume of 200 µL (*n* = 3 at each concentration). The cytotoxicity (as cell viability) was calculated using the formula mentioned previously [28].

### 2.12. Stability

Spray-dried powder with maximum in vitro aerosolization was stored in an open petri dish at 20 ± 2 °C/30 ± 2% RH (desiccator room condition) and 25 ± 2 °C /75% RH (elevated humidity condition) for one month to assess its physical (morphology and crystallinity), aerodynamic (aerosolization, MMAD and GSD) and chemical (drug content) stability using the methods mentioned above.

### 2.13. Statistical Analysis

Instat GraphPad Prism software (version 4.00; GraphPad Software, San Diego, CA, USA) was used for the statistical analyses (one-way analysis of variance (ANOVA) with the Student–Newman–Keuls test (compare all pairs) of the data. The level of significance was *P* < 0.05. All data are expressed as means ± standard deviations.

## 3. Results and Discussion

### 3.1. Process Yield and Drug Content

Spray-dried bedaquiline-only powder (SD-B) had a process yield of 36% and bedaquiline co-spray dried with l-leucine (SD-BL) had 48%. The low process yield of SD-B was due to the higher deposition of this powder on the cyclone walls which was apparent during the production process. Addition of l-leucine in the bedaquiline formulation reduced the wall deposition and hence improved the process yield. The ability of l-leucine to improve the process yield has been reported previously [26,27,48]. The yield of SD-BL (48%) powder indicates the successful spray-drying of the prepared formulation [49]. The average content of bedaquiline in the spray-dried powders was very close (± 3.0%) to the theoretical content. The absence of any additional peak in the HPLC analysis indicates the chemical stability of the drug during spray-drying and absence of impurities in the spray-dried powders.

### 3.2. Minimal Inhibitory Concentration (MIC)

The MICs of bedaquiline and bedaquiline with l-leucine are shown in Table 1. While l-leucine did not have any detectable MIC value, the bedaquiline and bedaquiline with l-leucine had the same MIC value of 0.1 µg/mL against *M. tuberculosis* mc^2^6230. The MIC values are similar to the values reported in the literature [50].

### 3.3. Morphology and Particle Size

**Figure 1:**
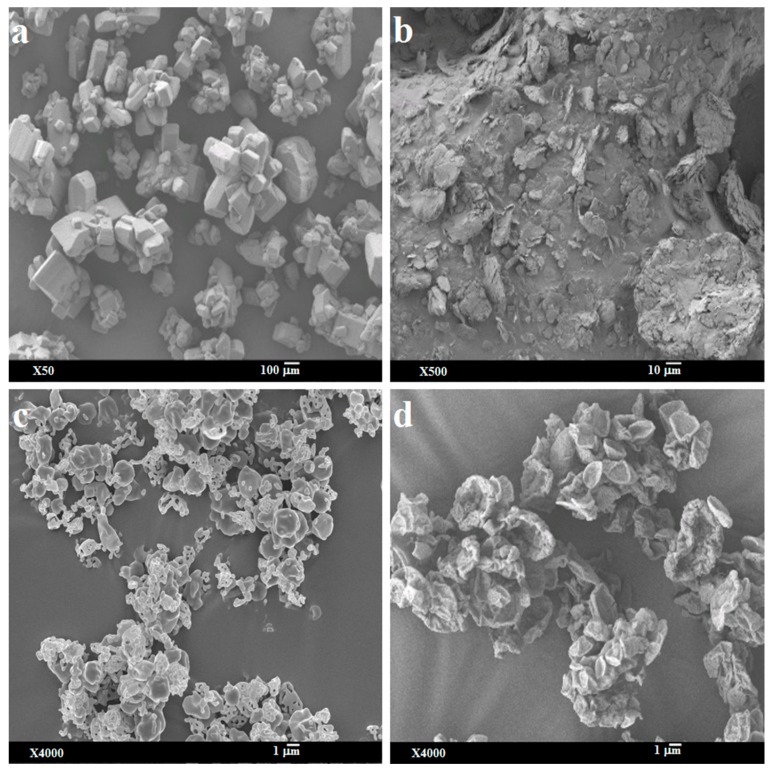
Representative scanning electron micrographs of: (**a**) supplied bedaquiline; (**b**) supplied l-leucine; (**c**) spray-dried bedaquiline-only and (**d**) spray-dried bedaquiline with 20% l-leucine.

The supplied bedaquiline particles were angular-shaped flakes with smooth surfaces, but l-leucine was irregularly shaped flakes with rough surfaces (Figure 1a,b). The geometric diameters of both the supplied materials were >10 µm (Figure 1a,b) which would not be suitable for deep lung delivery.

After spray-drying, the bedaquiline-only (SD-B) particles were mostly spherical, smooth dimpled, and aggregated with occasionally plate-like and porous (Figure 1c) forms, but the addition of l-leucine in bedaquiline formulation (SD-BL) produced plate-shaped particles with rough surfaces (Figure 1d). The differences in the shape and surface texture between SD-B and SD-BL particles could be due to the presence of surface active l-leucine in SD-BL. l-leucine precipitates on the droplet surface during the rapid drying process due to the differences in the solubility of components in the co-solvent systems and evaporation of co-solvent [20,44,51,52]. l-leucine adsorbs at the interface of the droplet to produce a surface film which eventually produces rough surfaces of the SD-BL particles. On the other hand, for SD-B formulation, there was no comparative precipitation of the component since only bedaquiline is present in the formulation. The geometric diameters of both SD-B and SD-BL particles were <5 µm (Figure 1) indicating the suitability for deep lung delivery [53]. However, the SD-B particles were more aggregated than SD-BL. The plate-shaped particles with rough surfaces in SD-BL might be advantageous for aerosolization [54,55].

### 3.4. Crystallinity

**Figure 2:**
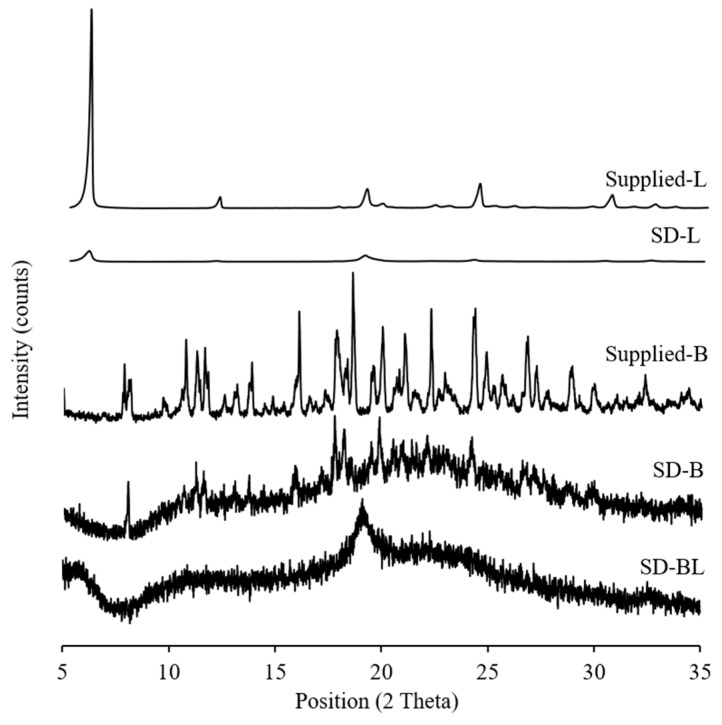
X-ray diffractograms of supplied materials and spray-dried powders (L and B mean l-leucine and bedaquiline; SD means spray-dried; BL means bedaquiline with 20% *w/w* of l-leucine).

Both supplied bedaquiline and l-leucine had sharp diffraction peaks in the X-ray diffractograms indicating the crystallinity of these materials (Figure 2). After spray drying, l-leucine remained crystalline but the peak intensities decreased. Halo diffraction peaks with reduced peak intensities were present in the spray-dried bedaquiline and bedaquiline with l-leucine powders suggesting the amorphous nature of these powders with traces of crystals (Figure 2) which is in agreement with the previous report that powders generally become amorphous in nature during spray-drying [20]. Amorphous materials are sensitive to amorphous–crystalline transition in the presence of moisture which affects their stability.

### 3.5. Thermal Analysis

**Figure 3:**
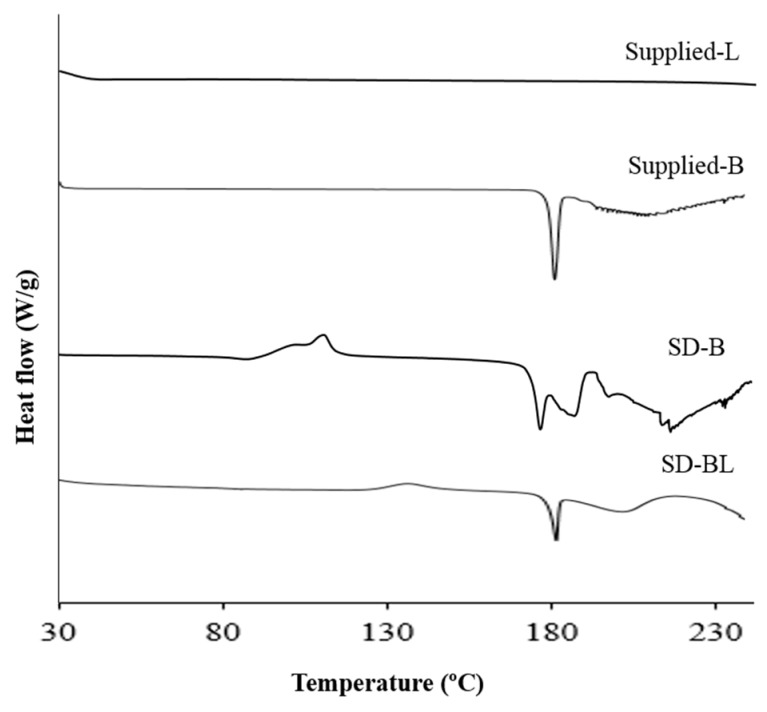
Differential scanning calorimetry (DSC) thermograms of supplied and spray-dried powders (L and B mean l-leucine and bedaquiline; SD means spray-dried; BL means bedaquiline with 20% *w/w* l-leucine).

**Figure 4:**
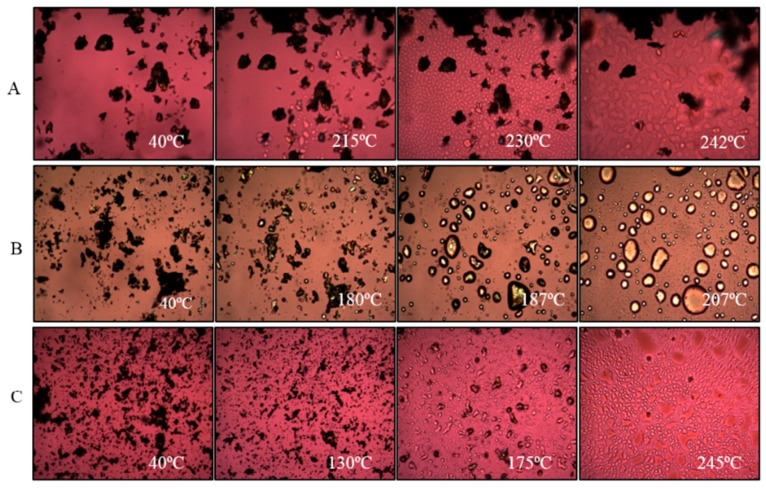
Representative hot-stage microscopy images for: (**A**) supplied l-leucine, (**B**) bedaquiline and (**C**) spray-dried bedaquiline with l-leucine.

Figure 3 and Figure 4 show the results of thermal analysis. Supplied l-leucine did not have any peak in the temperature range shown (Figure 3). However, supplied bedaquiline showed a sharp endothermic peak at 187 °C corresponding with its melting point and indicating the crystallinity of this material which is matching with the X-ray powder diffraction (XRPD) results (Figure 2).

The hot stage microscopy images for supplied bedaquiline (Figure 4B) also supports the findings of DSC and XRPD analyses. Supplied bedaquiline showed melting of the particles at 187 °C which continued until 207 °C (Figure 4B).

After spray-drying, bedaquiline-only (SD-B) powder showed a melting peak at around 180 °C and bedaquiline with l-leucine (SD-BL) powder showed a melting peak at around 185 °C (Figure 3). The peak in SD-B at around 190 °C can be attributed to a crystalline change of bedaquiline [56]. The broad peaks both in SD-B and SD-BL after 190 °C can be attributed to the decomposition of the materials. Similar to the DSC events, the hot-stage images of SD-BL particles showed signs of melting at 175 °C which gradually began to decompose at 183 °C which then continued (Figure 4C). The exothermic peaks in SD-B (at around 120 °C) and SD-BL (at around 130 °C) showed a possible solid–solid transition (amorphous to crystalline) of these materials.

### 3.6. Drug-Excipient Interaction Study

**Figure 5:**
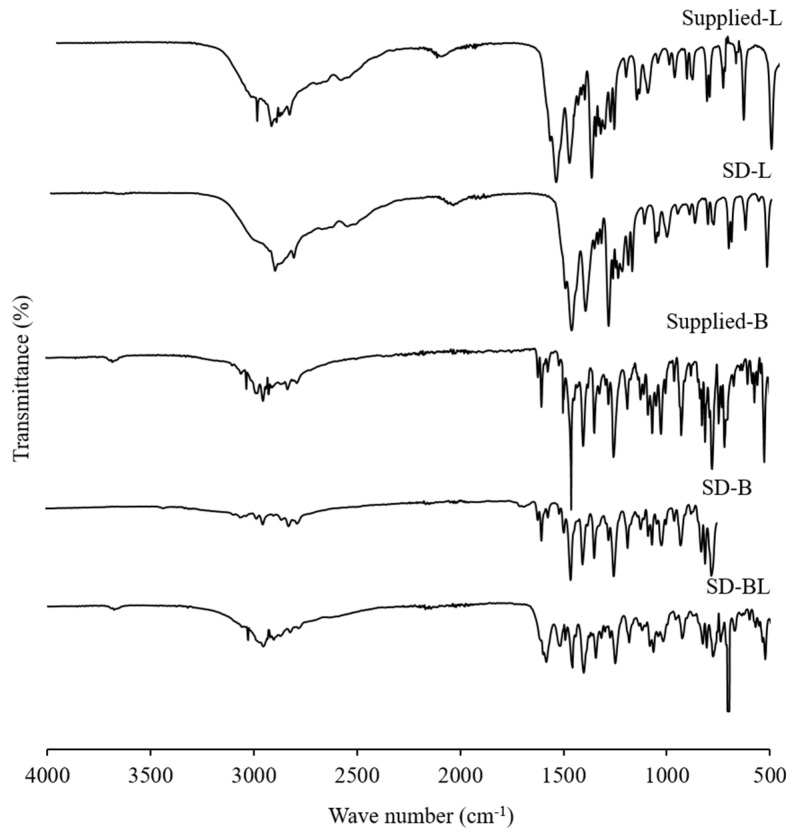
Attenuated total reflectance–Fourier transform infrared (ATR–FTIR) spectra of supplied l-leucine and bedaquiline and spray-dried powders of bedaquiline with and without l-leucine (L and B mean l-leucine and bedaquiline; SD means spray-dried; BL means bedaquiline with 20% *w/w* of l-leucine).

Figure 5 shows the ATR–FTIR spectra of the supplied and spray-dried powders. Similar characteristic peaks were observed in the ATR–FTIR spectra of both the supplied and spray-dried bedaquiline powders. The spectra of supplied bedaquiline powder showed major peaks for ether (at 1180 cm^−1^, 1067 cm^−1^ and 1058 cm^−1^), alcohol (3178 cm^−1^) and aromatic (3095 cm^−1^, 3053 cm^−1^, 3026 cm^−1^, 2974 cm^−1^, 2945 cm^−1^, 1616 cm^−1^, 1597 cm^−1^ and 1453 cm^−1^) functional groups (Figure 5).

After spray-drying with l-leucine (SD-BL) the peak positions were similar to the supplied bedaquiline with some bands of supplied l-leucine which is due to the lower percentage of l-leucine present in the formulation in comparison to bedaquiline. These findings confirm that there is no FTIR-detectable interaction between the components of the formulations during spray-drying.

### 3.7. Aerosolization

Table 2 shows the data for aerosolization performance of the spray-dried powders. Satisfactory recovery (between 84.1% and 91.4%) from the NGI was achieved during aerosolization studies. Both of the spray-dried powders had >80% of emitted dose (Table 2). The spray-dried bedaquiline-only powder had 31.3% FPF. Addition of l-leucine in the bedaquiline formulation improved the FPF to 74.4% (*P* < 0.05). Although it was reported that the aerosolization performance increases with the increase in l-leucine concentration in the formulation [23], a concentration beyond 20% *w/w* of l-leucine in the formulation does not improve the aerosolization performance and the physicochemical properties of the powders remain unchanged [57,58]. In this study, 20% *w/w* l-leucine was used since we previously found that 20% l-leucine was an appropriate concentration to improve the aerosolization performance and physical stability of powder formulations [35]. Since the fine particle fraction of bedaquiline with l-leucine formulation is higher than the bedaquiline alone formulation and both have the same MIC (Table 1), the use of bedaquiline with l-leucine formulation will be more effective.

The improved FPF in l-leucine-containing powder could be due to the change in morphology and aerodynamic properties. The addition of l-leucine produced plate-shaped particles with rough surfaces (Figure 1), and the mass median aerodynamic diameter was ≤ 2.4 µm (Table 2). On the other hand, SD-B particles were mostly spherical, smooth dimpled, and aggregated with occasional plate-like and porous (Figure 1c) forms, and the aerodynamic diameter was > 5.0 µm (Table 2). Previously it has been reported that particles with rough surfaces are better aerosolized than smooth surface particles, and the particles with < 3 µm aerodynamic diameter are better for deep lung delivery [30,31,59,60]. Particle aerodynamic diameter plays an important role in powder deposition at different regions of lungs by influencing deposition mechanisms. For particles with < 3 μm aerodynamic diameter, approximately 80% reach the lower airways and 50–60% the alveoli [30]. However, particle shape and surface roughness are important properties of dry powder particles which affect aerodynamic behaviour. Shape factor is directly used to calculate the aerodynamic diameter [30,31]. It is true that aerodynamic diameter plays an important role in powder deposition in the lungs. For example, for a similar aerodynamic size range, a pollen-shaped particle with a petal-like surface structure can improve the deposition more than other shapes [54]. Wrinkled and flake-shaped particles also had higher aerosolization than spherical and smooth surface particles due to their lower interparticulate contact [61]. On the other hand, particles with smooth surfaces showed higher flow property and aerosolization than similar sized particles with rough surfaces [62]. In reality, for similar size particles improved aerosolization was reported for both rough and smooth surfaces [54,62,63] indicating there is no clear relationship between the surface texture and aerosolization performance of DPI. The GSD values of the spray-dried particles were between 2.0 and 2.7 (Table 2), indicating the unimodal particle size distribution [64].

**Figure 6:**
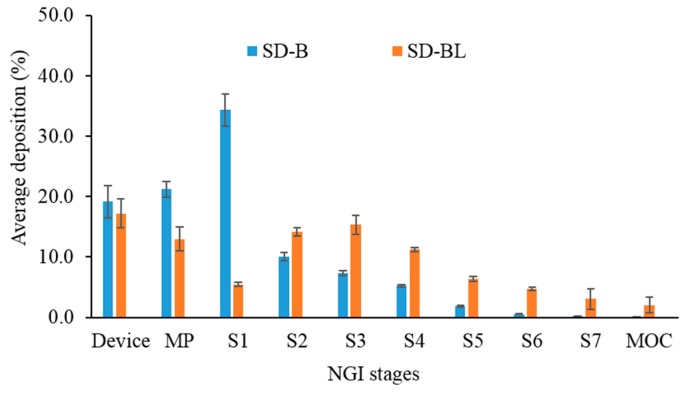
Deposition behaviour of spray-dried bedaquiline-only (SD-B) and bedaquiline with 20% *w/w* l-leucine (SD-BL) powders on different stages of next-generation impactor (NGI) (MP: mouthpiece, S1–S7 represent stages 1 to 7; MOC: micro-orifice collector, error bars represent standard deviations, *n* = 3).

In comparison to SD-B, the deposition of bedaquiline from SD-BL powder was higher on stages 2 to 7 and MOC (Figure 6) (*P* < 0.05). However, SD-B had higher depositions in MP and S1 than SD-BL, which has contributed to the higher FPF of SD-BL powder than SD-B (*P* < 0.05). Since SD-BL had the highest aerosolization performance, we proceeded with this powder for stability.

### 3.8. Stability

The results for aerodynamic parameters of the stored powder (SD-BL) are shown in Table 3. In comparison to the initial state, after one month storage at both desiccator and elevated humidity conditions, no significant difference was found in the FPF (*P* > 0.05). The MMAD and GSD values also remained unchanged after storage. However, ED was significantly decreased after storage at both conditions (*P* < 0.05) and the reason for this is not clear.

**Figure 7:**
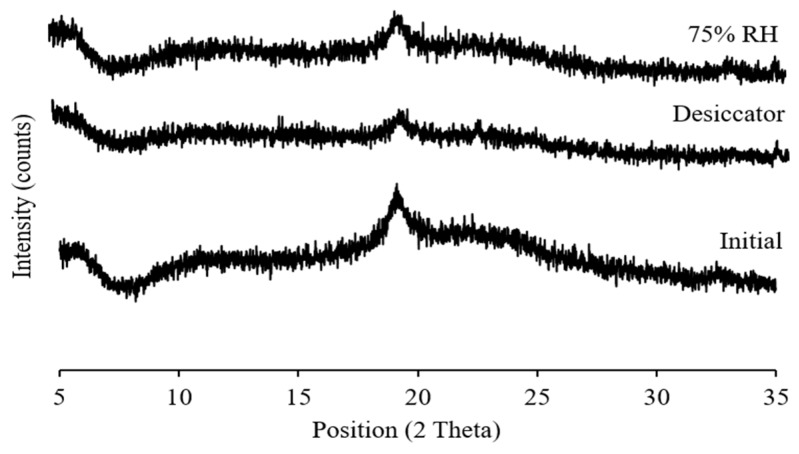
X-ray diffractograms of spray-dried bedaquiline with 20% *w/w* of l-leucine powder after one month’s storage at 30 ± 2% RH and ambient room temperature (in desiccator) and at 75 ± 2% RH and 25 ± 2 °C (elevated condition) (initial data from Figure 2 was used for comparison).

**Figure 8:**
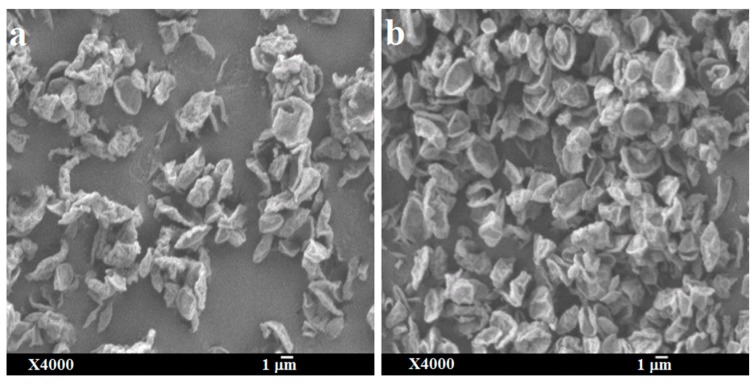
Scanning electron micrographs of spray-dried bedaquiline with 20% *w/w* of l-leucine powder after one month’s storage at two different conditions: (**a**) at 30 ± 2% RH and ambient room temperature (in desiccator) and (**b**) at 75 ± 2% RH and 25 ± 2 °C (elevated condition).

The physical state and particle morphology of the powder remained almost unchanged after one month’s storage at both desiccator and elevated humidity conditions (Figure 7 and Figure 8). After storage, the slight differences in the XRPD without a clear sign of crystallization indicates that the powder remained amorphous with an intuitive sign of changes which might be clearly visible after long-term storage of this powder. No degradation peaks were observed during HPLC analysis indicating the chemical stability of the stored powder. Previously it has been reported that l-leucine prevents moisture-induced deterioration in aerosolization and protects powder stability [65,66]. While no significant changes in aerosolization, morphology and crystallinity of SD-BL were observed during one month’s storage, it is not unlikely to have some changes during a longer-term storage (more than a month).

### 3.9. Cytotoxicity Studies

**Figure 9:**
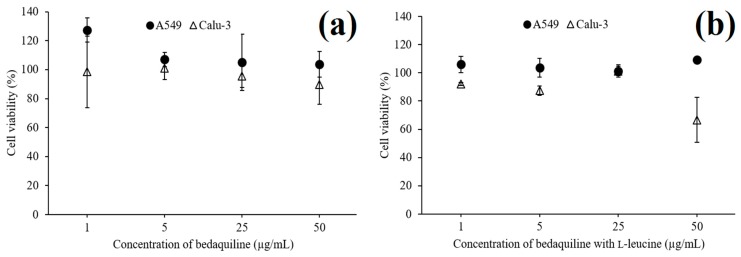
Cytotoxicity studies of spray-dried bedaquiline-only (**a**) and bedaquiline with l-leucine (**b**) powders using A549 cells and Calu-3 cells (data are means ± standard deviations, *n* = 3).

Spray-dried bedaquiline-only (SD-B) powder (Figure 9a) had no cytotoxic effects on A549 cells (>99% cell viability) and Calu-3 cells (>89% cell viability) up to the concentration (50 μg/mL) evaluated indicating the safety of the powder for inhalation delivery.

On the other hand, although l-leucine containing bedaquiline powder showed no toxicity on A549 cells at 50 μg/mL, for the same concentration the powder was toxic to Calu 3 cells (< 67% cell viability) (Figure 9b). This formulation was safe for Calu-3 cells up to a concentration of 25 μg/mL. The greater cytotoxicity of SD-BL powder than the SD-B could be due to the enhanced permeability of SD-BL powder in the presence of surface active l-leucine [67,68].

## 4. Conclusions

Inhalable dry powder particles of bedaquiline with high aerosolization efficiency were successfully produced using a spray-drying technique. The powders were amorphous in nature. The aerosolization efficiency of l-leucine containing bedaquiline powder (%FPF: 74.4) was higher than that of spray-dried bedaquiline-only powder (%FPF: 31.3) which is possibly due to the change in surface morphology and aerodynamic diameter. l-leucine containing powder particles were plate-shaped with rough surfaces and had a favourable aerodynamic diameter (≤ 2.4 µm) but the bedaquiline-only powder was spherical, smooth dimpled, and aggregated with occasionally plate-like and porous and >5.0 µm in size. The aerosolization, morphology and crystallinity of l-leucine containing bedaquiline powder were stable during one month’s storage at both desiccator and elevated humidity conditions. The powders were also non-toxic to the A549 respiratory cell-line at drug concentration up to 50 µg/mL and were potent inhibitors of *M. tuberculosis* growth. Further research is required to test the long-term stability, efficacy against infected animal models, and safety of the prepared formulations on animal models.

## Figures and Tables

**Table 1:** Minimum inhibitory concentrations (MICs) of bedaquiline and bedaquiline with l-leucine formulations against *Mycobacterium tuberculosis* mc^2^6230.

Compound	MIC (µg/mL)
Bedaquiline	0.1
Bedaquiline with l-leucine	0.1
l-leucine	ND

MICs were determined using a microtitre plate assay in biological triplicate at two-fold dilutions over the range 0.02–10 µg/mL. *M. tuberculosis* mc^2^6230 cultures were incubated with the compounds for 5 days before resazurin was added and MICs obtained. ND: not detected.

**Table 2:** Aerosolization performance of the spray-dried bedaquiline (with or without l-leucine) powders.

Formulation	SD-B	SD-BL
Recovery (%)	84.1 ± 0.6	91.4 ± 1.5
Emitted dose, ED (%)	80.8 ± 2.7	81.6 ± 3.2
Fine particle fraction, FPF (%ED)	31.3 ± 2.5	74.4 ± 4.3
Fine particle fraction, FPF (%RD)	25.3 ± 1.2	60.8 ± 5.8
Mass median aerodynamic diameter, MMAD (µm)	5.9 ± 0.1	2.4 ± 0.2
Geometric standard deviation, GSD	2.7 ± 0.1	2.0 ± 0.1

Note: SD means spray-dried; B and L mean bedaquiline and l-leucine, respectively. SD-BL means bedaquiline spray-dried powder with 20% *w/w* of l-leucine. RD means recovered dose. Data are means ± intra-batch standard deviations (*n* = 3).

**Table 3:** Aerosolization performance of the spray-dried bedaquiline (with 20% *w/w* of l-leucine) powder after one-month storage at 30 ± 2% relative humidity (RH) and ambient room temperature (in desiccator) and at 75 ± 2% RH and 25 ± 2 °C (elevated condition).

Conditions	Initial	Desiccator	75% RH
Recovery (%)	91.4 ± 1.5	89.0 ± 4.0	102.0 ± 3.0
ED (%)	81.6 ± 3.2	70.0 ± 1.0	75.0 ± 1.0
FPF (%)	74.4 ± 4.3	72.0 ± 5.0	63.0 ± 7.0
MMAD (µm)	2.4 ± 0.2	2.9 ± 0.4	3.1 ± 0.5
GSD	2.0 ± 0.1	2.1 ± 0.1	2.2 ± 0.1

Note: Data are means ± intra-batch standard deviations (*n* = 3). ED: emitted dose; FPF: fine particle fraction; MMAD: mass median aerodynamic diameter; GSD: geometric standard deviation.

## References

[1] Global Tuberculosis Report 2018. https://www.who.int/tb/publications/global_report/en/.

[2] Goldman R.C., Plumley K.V., Laughon B.E. (2007). The evolution of extensively drug resistant tuberculosis (XDR-TB): History, status and issues for global control. Infect. Disord. Drug Targets.

[3] Rawal T., Butani S. (2016). Combating tuberculosis infection: A forbidding challenge. Indian J. Pharm. Sci..

[4] Andries K., Verhasselt P., Guillemont J., Göhlmann H.W., Neefs J.M., Winkler H., Van Gestel J., Timmerman P., Zhu M., Lee E. (2005). A diarylquinoline drug active on the ATP synthase of Mycobacterium tuberculosis. Science.

[5] Cox E., Laessig K. (2014). FDA approval of bedaquiline—the benefit–risk balance for drug-resistant tuberculosis. N. Eng. J. Med..

[6] Huitric E., Verhasselt P., Andries K., Hoffner S.E. (2007). In vitro antimycobacterial spectrum of a diarylquinoline ATP synthase inhibitor. Antimicrob. Agents Chemother..

[7] Ibrahim M., Andries K., Lounis N., Chauffour A., Truffot-Pernot C., Jarlier V., Veziris N. (2007). Synergistic activity of R207910 combined with pyrazinamide against murine tuberculosis. Antimicrob. Agents Chemother..

[8] Field S.K. (2015). Bedaquiline for the treatment of multidrug-resistant tuberculosis: Great promise or disappointment?. Ther. Adv. Chronic Dis..

[9] Diacon A.H., Pym A., Grobusch M., Patientia R., Rustomjee R., Page-Shipp L., Pistorius C., Krause R., Bogoshi M., Churchyard G. (2009). The diarylquinoline TMC207 for multidrug-resistant tuberculosis. N. Eng. J. Med..

[10] Diacon A.H., Donald P.R., Pym A., Grobusch M., Patientia R.F., Mahanyele R., Bantubani N., Narasimooloo R., De Marez T., Van Heeswijk R. (2012). Randomized pilot trial of eight weeks of bedaquiline (TMC207) treatment for multidrug-resistant tuberculosis: Long-term outcome, tolerability, and effect on emergence of drug resistance. Antimicrob. Agents Chemother..

[11] Diacon A.H., Pym A., Grobusch M.P., de Los Rios J.M., Gotuzzo E., Vasilyeva I., Leimane V., Andries K., Bakare N., De Marez T. (2014). Multidrug-resistant tuberculosis and culture conversion with bedaquiline. N. Eng. J. Med..

[12] Mbuagbaw L., Guglielmetti L., Hewison C., Bakare N., Bastard M., Caumes E., Fréchet-Jachym M., Robert J., Veziris N., Khachatryan N. (2019). Outcomes of Bedaquiline Treatment in Patients with Multidrug-Resistant Tuberculosis. Emerg. Infect. Dis..

[13] Jones J., Mudaly V., Voget J., Naledi T., Maartens G., Cohen K. (2019). Adverse drug reactions in South African patients receiving bedaquiline-containing tuberculosis treatment: An evaluation of spontaneously reported cases. BMC Infect. Dis..

[14] Sacks L.V., Pendle S., Orlovic D., Andre M., Popara M., Moore G., Thonell L., Hurwitz S. (2001). Adjunctive salvage therapy with inhaled aminoglycosides for patients with persistent smear-positive pulmonary tuberculosis. Clin. Infect. Dis..

[15] Sermet-Gaudelus I., Le Cocguic Y., Ferroni A., Clairicia M., Barthe J., Delaunay J.P., Brousse V., Lenoir G. (2002). Nebulized antibiotics in cystic fibrosis. Pediatric Drugs.

[16] Garcia-Contreras L., Muttil P., Fallon J.K., Kabadi M., Gerety R., Hickey A.J. (2012). Pharmacokinetics of sequential doses of capreomycin powder for inhalation in guinea pigs. Antimicrob. Agents Chemother..

[17] Garcia-Contreras L., Sung J., Ibrahim M., Elbert K., Edwards D., Hickey A. (2015). Pharmacokinetics of inhaled rifampicin porous particles for tuberculosis treatment: Insight into rifampicin absorption from the lungs of guinea pigs. Mol. Pharm..

[18] Das S., Tucker I., Stewart P. (2015). Inhaled dry powder formulations for treating tuberculosis. Curr. Drug Deliv..

[19] Masters K. (1991). Spray Drying Handbook.

[20] Vehring R. (2008). Pharmaceutical particle engineering via spray drying. Pharm. Res..

[21] Chew N.Y., Shekunov B.Y., Tong H.H., Chow A.H., Savage C., Wu J., Chan H.K. (2005). Effect of amino acids on the dispersion of disodium cromoglycate powders. J. Pharm. Sci..

[22] Li H.Y., Seville P.C., Williamson I.J., Birchall J.C. (2005). The use of amino acids to enhance the aerosolisation of spray-dried powders for pulmonary gene therapy. J. Gene Med..

[23] Seville P.C., Learoyd T.P., Li H.Y., Williamson I.J., Birchall J.C. (2007). Amino acid-modified spray-dried powders with enhanced aerosolisation properties for pulmonary drug delivery. Powder Technol..

[24] Sou T., Orlando L., McIntosh M.P., Kaminskas L.M., Morton D.A. (2011). Investigating the interactions of amino acid components on a mannitol-based spray-dried powder formulation for pulmonary delivery: A design of experiment approach. Int. J. Pharm..

[25] Boraey M.A., Hoe S., Sharif H., Miller D.P., Lechuga-Ballesteros D., Vehring R. (2013). Improvement of the dispersibility of spray-dried budesonide powders using leucine in an ethanol–water cosolvent system. Powder Technol..

[26] Sou T., McIntosh M.P., Kaminskas L.M., Prankerd R.J., Morton D.A. (2013). Designing a multicomponent spray-dried formulation platform for pulmonary delivery of biomacromolecules: The effect of polymers on the formation of an amorphous matrix for glassy state stabilization of biomacromolecules. Dry. Technol..

[27] Sou T., Kaminskas L.M., Nguyen T.H., Carlberg R., McIntosh M.P., Morton D.A. (2013). The effect of amino acid excipients on morphology and solid-state properties of multi-component spray-dried formulations for pulmonary delivery of biomacromolecules. Eur. J. Pharm. Biopharm..

[28] Momin M.A.M., Sinha S., Tucker I.G., Doyle C., Das S.C. (2017). Dry powder formulation of kanamycin with enhanced aerosolization efficiency for drug-resistant tuberculosis. Int. J. Pharm..

[29] Rawal T., Patel S., Butani S. (2018). Chitosan nanoparticles as a promising approach for pulmonary delivery of bedaquiline. Eur. J. Pharm. Sci..

[30] Heyder J.J.G.C.F.W., Gebhart J., Rudolf G., Schiller C.F., Stahlhofen W. (1986). Deposition of particles in the human respiratory tract in the size range 0.005–15 μm. J. Aerosol Sci..

[31] Momin M.A.M., Tucker I.G., Das S.C. (2018). High dose dry powder inhalers to overcome the challenges of tuberculosis treatment. Int. J. Pharm..

[32] Eedara B.B., Tucker I.G., Das S.C. (2016). Phospholipid-based pyrazinamide spray-dried inhalable powders for treating tuberculosis. Int. J. Pharm..

[33] Eedara B.B., Rangnekar B., Doyle C., Cavallaro A., Das S.C. (2018). The influence of surface active l-leucine and 1,2-dipalmitoyl-*sn*-glycero-3-phosphatidylcholine (DPPC) in the improvement of aerosolization of pyrazinamide and moxifloxacin co-spray dried powders. Int. J. Pharm..

[34] Momin M.A.M., Tucker I.G., Doyle C.S., Denman J.A., Sinha S., Das S.C. (2018). Co-spray drying of hygroscopic kanamycin with the hydrophobic drug rifampicin to improve the aerosolization of kanamycin powder for treating respiratory infections. Int. J. Pharm..

[35] Momin M.A.M., Sinha S., Tucker I.G., Das S.C. (2019). Carrier-free combination dry powder inhaler formulation of ethionamide and moxifloxacin for treating drug-resistant tuberculosis. Drug Devel. Ind. Pharm..

[36] Shetty N., Park H., Zemlyanov D., Mangal S., Bhujbal S., Zhou Q.T. (2018). Influence of excipients on physical and aerosolization stability of spray dried high-dose powder formulations for inhalation. Int. J. Pharm..

[37] Mangal S., Nie H., Xu R., Guo R., Cavallaro A., Zemlyanov D., Zhou Q.T. (2018). Physico-chemical properties, aerosolization and dissolution of co-spray dried azithromycin particles with l-leucine for inhalation. Pharm. Res..

[38] Jong T., Li J., Morton D.A., Zhou Q.T., Larson I. (2016). Investigation of the changes in aerosolization behavior between the jet-milled and spray-dried colistin powders through surface energy characterization. J. Pharm. Sci..

[39] ICH Validation of Analytical Procedures: Methodology. Proceedings of the International Conference on Harmonization.

[40] Alffenaar J.W.C., Bolhuis M., van Hateren K., Sturkenboom M., Akkerman O., de Lange W., Greijdanus B., van der Werf T., Touw D. (2015). Determination of bedaquiline in human serum using liquid chromatography-tandem mass spectrometry. Antimicrob. Agents Chemother..

[41] Santoso K.T., Menorca A., Cheung C.Y., Cook G.M., Stocker B.L., Timmer M.S. (2019). The synthesis and evaluation of quinolinequinones as anti-mycobacterial agents. Bioorg. Med. Chem..

[42] Bardarov S., Bardarov Jr S., Pavelka Jr M.S., Sambandamurthy V., Larsen M., Tufariello J., Chan J., Hatfull G., Jacobs Jr W.R. (2002). Specialized transduction: An efficient method for generating marked and unmarked targeted gene disruptions in Mycobacterium tuberculosis, M. bovis BCG and M. smegmatis. Microbiol..

[43] Sambandamurthy V.K., Wang X., Chen B., Russell R.G., Derrick S., Collins F.M., Morris S.L., Jacobs Jr W.R. (2002). A pantothenate auxotroph of Mycobacterium tuberculosis is highly attenuated and protects mice against tuberculosis. Nat. Med..

[44] Momin M.A.M., Tucker I.G., Doyle C.S., Denman J.A., Das S.C. (2018). Manipulation of spray-drying conditions to develop dry powder particles with surfaces enriched in hydrophobic material to achieve high aerosolization of a hygroscopic drug. Int. J. Pharm..

[45] de Boer A.H., Chan H.K., Price R. (2012). A critical view on lactose based drug formulation and device studies for dry powder inhalation: Which are relevant and what interactions to expect?. Adv. Drug Deliv. Rev..

[46] Chan J.G., Duke C.C., Ong H.X., Chan J.C., Tyne A.S., Chan H.K., Britton W.J., Young P.M., Traini D. (2014). A novel inhalable form of rifapentine. J. Pharm. Sci..

[47] Chan J.G.Y., Chan H.K., Prestidge C.A., Denman J.A., Young P.M., Traini D. (2013). A novel dry powder inhalable formulation incorporating three first-line anti-tubercular antibiotics. Eur. J. Pharm. Biopharm..

[48] Chen K.H., Mueannoom W., Gaisford S., Kett V.L. (2012). Investigation into the effect of varying l-leucine concentration on the product characteristics of spray-dried liposome powders. J. Pharm. Pharmacol..

[49] Adhikari B., Howes T., Lecomte D., Bhandari B.R. (2005). A glass transition temperature approach for the prediction of the surface stickiness of a drying droplet during spray drying. Powder Technol..

[50] Lopez B., Siqueira de Oliveira R., Pinhata J.M., Chimara E., Pacheco Ascencio E., Puyén Guerra Z.M., Wainmayer I., Simboli N., Del Granado M., Palomino J.C. (2018). Bedaquiline and linezolid MIC distributions and epidemiological cut-off values for Mycobacterium tuberculosis in the Latin American region. J. Antimicrob. Chemother..

[51] Bosquillon C., Rouxhet P.G., Ahimou F., Simon D., Culot C., Préat V., Vanbever R. (2004). Aerosolization properties, surface composition and physical state of spray-dried in powders. J. Controlled Rel..

[52] Kim E.H.J., Dong Chen X., Pearce D. (2003). On the mechanisms of surface formation and the surface compositions of industrial milk powders. Dry. Technol..

[53] Zeng X.M., Martin G.P., Marriott C. (1995). The controlled delivery of drugs to the lung. Int. J. Pharm..

[54] Hassan M.S., Lau R.W.M. (2009). Effect of particle shape on dry particle inhalation: Study of flowability, aerosolization, and deposition properties. AAPS Pharmscitech..

[55] Das S.C., Behara S.R.B., Morton D.A., Larson I., Stewart P.J. (2013). Importance of particle size and shape on the tensile strength distribution and de-agglomeration of cohesive powders. Powder Technol..

[56] Xu J., Zhang L., Zhang X.Y., Wang X.Z., Chai J., Luo H.Y., Yang Z.Q. (2018). Crystal forms of bedaquiline fumarate and preparation methods therefor. U.S. Patent.

[57] Li L., Sun S., Parumasivam T., Denman J.A., Gengenbach T., Tang P., Mao S., Chan H.K. (2016). l-Leucine as an excipient against moisture on in vitro aerosolization performances of highly hygroscopic spray-dried powders. Eur. J. Pharm. Biopharm..

[58] Mangal S., Meiser F., Tan G., Gengenbach T., Denman J., Rowles M.R., Larson I., Morton D.A. (2015). Relationship between surface concentration of L-leucine and bulk powder properties in spray dried formulations. Eur. J. Pharm. Biopharm..

[59] Adi H., Traini D., Chan H.K., Young P.M. (2008). The influence of drug morphology on aerosolisation efficiency of dry powder inhaler formulations. J. Pharm. Sci..

[60] Jackson B.C., Bennett D.J., Bartus R.T., Emerich D.F. (2015). Pulmonary Delivery for Levodopa. U.S. Patent.

[61] Zijlstra G.S., Hinrichs W.L., de Boer A.H., Frijlink H.W. (2004). The role of particle engineering in relation to formulation and de-agglomeration principle in the development of a dry powder formulation for inhalation of cetrorelix. Eur. J. Pharm. Sci..

[62] El-Sabawi D., Edge S., Price R., Young P.M. (2006). Continued investigation into the influence of loaded dose on the performance of dry powder inhalers: Surface smoothing effects. Drug Dev. Ind. Pharm..

[63] Chew N.Y., Tang P., Chan H.K., Raper J.A. (2005). How much particle surface corrugation is sufficient to improve aerosol performance of powders?. Pharm. Res..

[64] Glover W., Chan H.K., Eberl S., Daviskas E., Verschuer J. (2008). Effect of particle size of dry powder mannitol on the lung deposition in healthy volunteers. Int. J. Pharm..

[65] Chan J.G.Y., Tyne A.S., Pang A., Chan H.K., Young P.M., Britton W.J., Duke C.C., Traini D. (2014). A rifapentine-containing inhaled triple antibiotic formulation for rapid treatment of tubercular infection. Pharm. Res..

[66] Yu J., Chan H.K., Gengenbach T., Denman J.A. (2017). Protection of hydrophobic amino acids against moisture-induced deterioration in the aerosolization performance of highly hygroscopic spray-dried powders. Eur. J. Pharm. Biopharm..

[67] Mitra R., Pezron I., Li Y., Mitra A.K. (2001). Enhanced pulmonary delivery of insulin by lung lavage fluid and phospholipids. Int. J. Pharm..

[68] Codrons V., Vanderbist F., Ucakar B., Préat V., Vanbever R. (2004). Impact of formulation and methods of pulmonary delivery on absorption of parathyroid hormone (1–34) from rat lungs. J. Pharm. Sci..

